# Time to Diagnose Endometriosis: Current Status, Challenges and Regional Characteristics—A Systematic Literature Review

**DOI:** 10.1111/1471-0528.17973

**Published:** 2024-10-07

**Authors:** Pauline De Corte, Moritz Klinghardt, Sophia von Stockum, Klaas Heinemann

**Affiliations:** ^1^ Berlin Center for Epidemiology and Health Research Berlin Germany

**Keywords:** diagnostic delay, endometriosis, time to diagnosis

## Abstract

**Background:**

Endometriosis diagnosis reportedly faces delays of up to 10 years. Despite growing awareness and improved guidelines, information on the current status is limited.

**Objectives:**

To systematically assess the published evidence on the status of time to diagnosis in individuals with endometriosis, with respect to the definition of time to diagnosis, geographical location and patient characteristics.

**Search Strategy:**

MEDLINE (via PubMed) and Embase were searched for publications reporting time to diagnosing endometriosis since 2018. No restrictions to population or comparators were applied. All publications were screened by two independent reviewers.

**Selection Criteria:**

Search results were limited to primary publications of randomised controlled trials, non‐randomised trials and observational studies. Case reports, secondary publications and grey literature were excluded. No restrictions were made regarding language, provided that an English title and abstract were available.

**Data Collection and Analysis:**

Publications were assessed with respect to time to diagnosis, diagnostic methods, study type, study country and potential bias.

**Main Results:**

The 17 publications eligible for inclusion in this literature review were all observational studies. The publications reported diagnosis times between 0.3 and 12 years, with variations depending on the definition of time to diagnosis (overall, primary, or clinical), geographical location and characteristics of the included study population. Evidence was of poor to good quality overall.

**Conclusions:**

Diagnostic delay is still present, primarily driven by physicians, and this review underscores the need for standardised definitions, increased awareness and targeted diagnostic interventions.

## 1 Introduction

Endometriosis research has gained considerable interest over the last decades. Despite growing interest, numerous challenges still remain, including the lack of universal diagnostic criteria, the difficulty of identifying a definite diagnosis due to the diverse symptomatology and diagnostic challenges, the ongoing normalisation of underlying endometriosis symptoms by both the health care provider and patient and many more [1, 2, 3]. These factors contribute to a delay between the onset of first symptoms and a definite diagnosis, ultimately delaying effective treatment. The delay can have profound implications for patients, contributing to potentially worsened symptoms [4, 5], impaired quality of life [6, 7] and increased healthcare costs [8]. As the diagnostic delay seems to be a catalyst for many challenges in the endometriosis research field currently investigated, reducing the diagnostic delay is key.

Diagnostic delay in endometriosis is a well‐known phenomenon, with studies consistently reporting prolonged intervals of up to 7–10 years between the symptom onset and confirmed diagnosis [8]. A 1997 study observed a decreased time to diagnosis from 9.21 to 4.63 years over 15 years [5]. A recent study in the United States noted a mean time from symptom onset to diagnosis of 4.4 years, suggesting a potentially shorter diagnostic journey in contemporary healthcare settings [9]. In recent years, efforts have been made to raise awareness about endometriosis and reduce delays through initiatives such as awareness campaigns [10], patient advocacy groups [11] and educational programs for healthcare professionals [12]. Numerous (inter)national organisations have published guidelines on the diagnosis of endometriosis: the American College of Obstetricians and Gynecologists [13], the European Society of Human Reproduction and Embryology [14], the Royal Australian and New Zealand College of Obstetricians and Gynaecologists [15] and the UK National Institute for Health and Care Excellence [16]. These guidelines aim for increased awareness among healthcare providers about the diagnostic criteria of endometriosis and thus may lead to earlier recognition and referral of affected individuals for specialist evaluation. Additionally, the standardisation of diagnostic protocols outlined in these guidelines may streamline the diagnostic process, reducing unnecessary delays caused by variations in clinical practice.

Given these numerous advancements, the increased awareness and research interest in the field of endometriosis, our aim was to assess the current status of time to diagnosis, focusing on studies published from 2018 onwards. More specifically, we aimed to quantify the current time to diagnosis in different geographical regions and population groups (e.g., comorbidities, symptoms, ethnicity and gender).

## 2 Methods

### 2.1 Literature Search

A pre‐specified protocol (PROSPERO ID: CRD42023453141) was followed for this study which adhered to the current Preferred Reporting Items for Systematic Reviews and Meta‐Analyses (PRISMA) statement (Appendix S2). A comprehensive literature search using MEDLINE (via PubMed) and Embase was performed to identify (non‐) randomised trials, observational longitudinal and cross‐sectional studies, case reports and case series from January 1, 2018 to May 16, 2023, the day of conducting the search. By focusing on studies published from 2018 onwards, we aimed to capture the most current data, reflecting the contemporary clinical environment and patient experience with respect to time to diagnosis. The complete search string is provided as Supporting Information (Appendix S1). In summary, a combination of MeSH (medical subject headings) or Emtree index terms and free text search terms were used to identify eligible publications.

For the scope of our search, all publications related to individuals diagnosed with endometriosis and reported information on diagnosis time were targeted. Identified publications were screened independently by two reviewers based on title and abstract, before conducting a full text assessment on the remaining publications. The following inclusion and exclusion criteria were applied for the search. Publications that focused on individuals of all ages diagnosed with endometriosis could be included. All methods for diagnoses were accepted (e.g., based on clinical symptoms, laparoscopy, etc.), provided that the diagnosis was confirmed by the treating physician. Publications were included regardless of interventions or comparators if they allowed an assessment of diagnosis time. Included publications were limited to primary publications (such as original research articles or conference abstracts), whereas secondary publications (e.g., narrative reviews, systematic reviews, or meta‐analyses), grey literature (e.g., government reports, graduate dissertations, unpublished clinical trials, etc.) and anecdotical evidence (e.g., case reports) were excluded. Articles of all languages were included, provided that an English title and abstract were available for screening of search results. Eligible publications in languages other than English were assessed with an automated translation using Google translate. Publications were included or excluded according to unanimous votes of both reviewers. Any discrepant findings were resolved in discussion.

**FIGURE 1:**
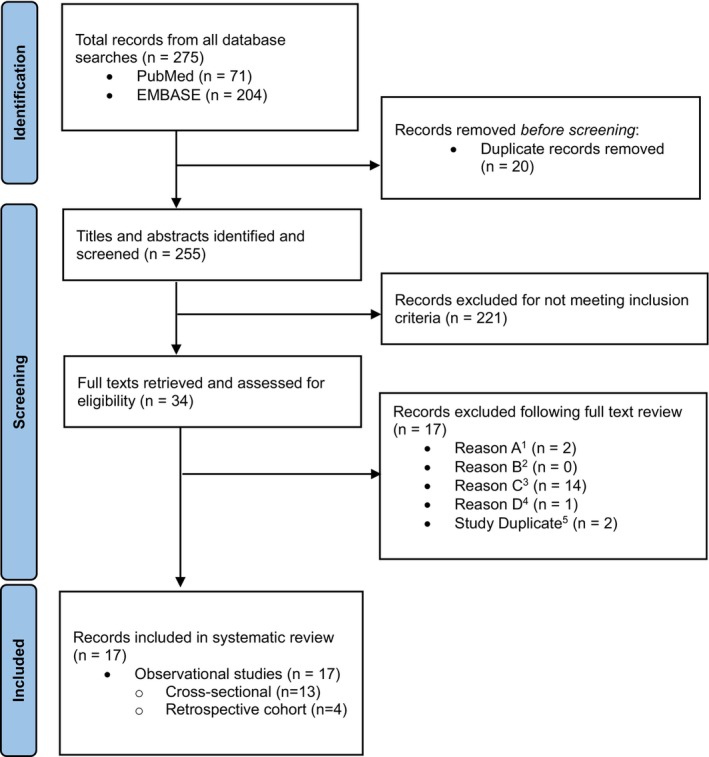
Flowchart of search process and study selection according to PRISMA guidelines. Multiple reasons allowed. ^1^A: Population (i.e., persons not diagnosed with endometriosis); ^2^B: Intervention (i.e., publications with and without interventions that do not allow an assessment of diagnosis time); ^3^C: Outcome (i.e., publications that do not allow an assessment of diagnosis time); ^4^D: Study type (i.e., case reports); ^5^Several records reporting on the same study results.

A flowchart depicting the screening process is provided in Figure 1. A qualitative and narrative synthesis of included publications was performed. The following information was extracted from each publication: reported diagnosis time, study design, overall sample size, study country/−ies, gender, population of interest, time of data collection, diagnostic methods and specialty of the diagnosing physician. Ethics approval was not required for the scope of the systematic review.

### 2.2 Bias Assessment

The risk of bias was critically assessed by two independent reviewers for a synoptic view and a subsequent discussion of bias across included publications. The following appraisal tools were used depending on the study type: the critical appraisal skills programme (CASP) tool [17] for cohort studies and the critical appraisal tool for cross‐sectional studies (AXIS) [18].

### 2.3 Presentation of Overall Time to Diagnosis

Mean and/or median time to diagnosis was presented as reported in single publications. In case only ranges were reported, mean years until diagnosis were calculated by first determining the midpoints of each range. Each midpoint was then multiplied by the number of participants in that range to obtain a weighted sum and was then divided by the total sample size.

### 2.4 Definition of Diagnostic Delay

The authors classified diagnosis time according to three definitions: (1) the time between the onset of first symptoms until diagnosis of endometriosis by a physician (i.e., overall diagnosis time), (2) the time between the onset of first symptoms until the first related physician visit (i.e., primary diagnosis time) and (3) the time between the first physician visit and diagnosis of endometriosis (i.e., clinical diagnosis time).

## 3 Results

### 3.1 Search Results

The literature search was performed in both PubMed and Embase on May 16, 2023. The used search strings (see Appendix S1) resulted in a total of 275 publications (PubMed: 71; Embase: 204). After excluding duplicate entries, 255 publications remained for abstract screening, of which 221 were excluded. Full texts of the remaining 34 publications were then screened again, of which 17 remained for data extraction. An overview of the screening process is provided in Figure 1 together with the main reasons for exclusion of full texts. A comprehensive list of all publications is given in Appendix S3 (Table S1).

### 3.2 General Findings

All 17 publications included in this review were observational studies and encompassed two design types, namely, cross‐sectional and retrospective cohort designs. The geographical distribution of included studies was confined to Western high‐income nations. Four were based in the United Kingdom [19, 20, 21], of which one also comprised Ireland [22], four in the United States [23, 24, 25, 26], three in Australia [27, 28, 29] and one each in Germany [30], France [31], Italy [32], Canada [33] and New Zealand [34]. One publication did not specify the geographical location [35]. The included sample sizes ranged from 49 to 11 793 individuals with a physician‐confirmed endometriosis diagnosis. A summarised overview of included publications is provided in Table 1. Methods used to diagnose endometriosis varied between publications and included laparoscopy, histological confirmation, surgical confirmation (surgery unspecified), empirical/clinical evaluation, physician‐suspected, imaging or not specified. In 13 out of 17 publications, the diagnostic confirmation method was self‐reported by the study participants.

**TABLE 1:** Summarised overview of included publications.

Author and year	Study type (data source)	Sample size	Time of data collection	Baseline characteristics available (Yes/No)?	Gender	Study country/−ies	Symptoms prior to diagnosis/at onset	Symptom profile included participants	Diagnosis methods	Overall diagnosis time	Primary diagnosis time	Clinical diagnosis time	Diagnosing physician specialty
Bullo et al. 2020	Observational cross‐sectional study (online survey)	*N* = 131	Not available	No	Female	UK and Ireland	Pelvic pain, menstruation‐related pain	Not specified	Method not specified, selection from the Language of Endo‐metriosis' social media platforms	Not available	Not available	8.6 years	Not available
Tewhaiti‐Smith et al. 2022	Observational cross‐sectional study (online survey)	*N* = 620	Mar‐May 2021	Yes (age, occupation, ethnicity, education, income, gyn history)	Female	Aotearoa New Zealand	Severe dysmenorrhoea (88.7%) Non‐cyclical pelvic pain (64.4%) Ovulation pain (46.1%) Chronic fatigue (46.3%) Cyclical/peri‐menstrual symptoms (40%) Deep dyspareunia (27.9%) Subfertility (7.3%)	Pelvic pain with periods in the last 3 months Occasionally (5.8%) Often (10.5%) Always (83.6%)	Laparo‐scopy	8.7 years	2.9 years (± 4.0) for those with endometriosis	5.8 (± 5.7) years Before 2005: 8.4 years (± 7.0) 2005–2012: 5.3 years (± 4.0) After 2012: 2.0 years (± 1.9)	Not specified
Markowitz et al. 2023	Observational cross‐sectional study (retrospective chart review)	*N* = 152	Jan 2017–Dec 2020	Yes (BMI)	Female	Not Available	Not specified	Not specified	Laparoscopy	Not Available	Not Available	Obese: 18.4 months (IQR 3.1–42.8) Overweight: 9.0 months (IQR 2.5–23.2) Normal and underweight: 3.8 months (IQR 1.1–17.0)	Physicians from an academic tertiary hospital
Nicolaus et al. 2020	Observational cross‐sectional study (postal survey)	*N* = 266	Jan 2016‐Dec 2017	Yes (age, gyn history)	Female	Germany	With infertility *N* = 76 Without infertility *N* = 106 Chronic pelvic pain (91.8%) Dyspareunia (53.3%) Dysmenorrhea (47.3%) Dyschezia (46.7%) Dysuria (24.7%)	Not specified	Histologically confirmed	< 1 year (13.5%–16.9%) 1–5 years (29.8%–31.0%) 6–10 years (12.7%–19.2%) > 10 years (37.5%–39.4%)	Not available	Not available	Not specified
Ghai et al. 2020	Observational cross‐sectional study (postal survey)	*N* = 101	2014	Yes (gyn history)	Female	UK	Menstrual cramps (73.3%) Trying to conceive > 12 months (48%)	Not specified	Laparoscopy with a confirmatory histology	Median time 8 years (Q1–Q3: 3–14) Women with rectovaginal endometriosis: Median time of 11 years (Q1–Q3: 4–16) Women with superficial endometriosis: median 5 years (Q1–Q3: 1–11) Women that experienced menstrual cramps during adolescence: median 11 years (Q1–Q3: 4.25–16) Women that did not experience menstrual cramps during adolescence: median 2 years (Q1–Q3: 1–5)	From GP to gynaecologist: 1 year (Q1–Q3: 0–4) From gynaecologist to diagnosis: 0 years (Q1–Q3: 0–1) The perceived attitudes of the health professional to pain also influenced the diagnosis. Women who felt their pain were not taken seriously by their GP experienced a twofold time from first symptom(s) to diagnosis (median 12, Q1–Q3: 5.25–15.75 vs. 6, Q1–Q3: 1–13) Those who felt that their gynaecologist did not acknowledge their symptoms experienced a median diagnosis time from their initial consultant to a diagnosis of 1 year (Q1–Q3: 0–4.5 vs. 0, Q1–Q3:0–1)	3 years (Q1–Q3: 0–10)	Gynaecologists
Karavadra et al. 2021	Observational cross‐sectional study (triphasic design: **online survey**, semi‐structured interviews, focus groups with healthcare workers)	*N* = 1252 replied to the online questionnaire *N* = 16 (took part in semi‐structured interviews) *N* = 15 (Healtcare workers participated in focus groups)	Not available	No	Female	UK	Not specified	Not specified	Not specified	5.5 years	Not available	Not available	Not specified
Armour et al. 2020	Observational cross‐sectional study (online survey)	*N* = 409 responses were received of which *N* = 340 have endometriosis *N* = 67 have chronic pelvic pain without endometriosis diagnosis	Feb 2017‐Apr 2017	Yes (age, ethnicity, relationship status, occupation, education, gyn history)	Not specified	Australia	Severe dysmenorrhea (89.4%) Deep Dyspareunia (32.3%) Pelvic pain (78.7%) Ovulation pain (46.7%) Cyclical or peri‐menstrual (38.1%) Infertility (7.6%) Chronic fatigue (38.8%)	Pelvic pain with periods in last 3 months (85.9%) Pelvic pain with intercourse (69.1%)	Surgically confirmed	8 years	2.9 years (± 4.4)	4.9 years (± 5.7)	Not specified
Whitfield et al. 2022	Observational cross‐sectional study (retrospective chart review)	*N* = 9413	N/A	No	Not specified	UK	Abdominal pain (61%) Mental health symptoms (34%) Irregular menstruation (32%)	Not specified	Method not specified (recorded diagnosis of endometriosis)	3.7 years Subgroups by age in paper	Not available	Not available	Not specified
Surrey et al. 2020	Observational cohort study (retrospective chart review)	*N* = 11 793 *n* = 4446 short delay (≤ 1 year) *n* = 3179 intermediate delay (1–3 years) *n* = 4168 long delay (3–5 years)	Jan 2004‐Jul 2016	Yes (age, origin, gyn history, comorbidities)	Not specified	US	Dyspareunia, pelvic pain, abdominal pain, dysmenorrhea, infertility	Not available	Method not specified (diagnosis based on medical claim for endometriosis in any position, ICD‐9‐CM/ICD‐10‐CM code 617.x/N80.x)	Mean: 763.9 ± 631.0 days (2.09 ± 1.77 years) Mean short delay: 90.2 days Mean intermediate delay: 733.4 days Mean long delay: 1505.9 days	Not available	Not available	Not specified
O'Hara et al. 2022	Observational cross‐sectional study (online survey)	*N* = 620 *n* = 601 responded to diagnostic delay question	Nov 2017‐Jan 2018	Yes (age, origin, education, relationship status, language, insurance, occupation, gyn history)	Female	Australia	GSWH survey comprising list of symptoms*	Pelvic pain with periods (65.8%), pelvic pain with intercourse (61.1%), pain at other times (82.7%)	Surgical	Mean: 6.4 ± 5.8 years	Not available	Not available	Not specified
Bontempo et al. 2020	Observational cross‐sectional study (online survey)	*N* = 758 *n* = 601 completed the survey	April 2017	Yes (age, race, marital status, education, income, origin, gyn history)	Not specified	US	List of symptoms**	Infertility (47.1%), average worst pain level (0–100) 88.6	Surgical (*self‐reported*)	Mean: 8.6 ± 6.5 years	Not available	Not available	Not specified
Singh et al. 2020	Observational cross‐sectional study (online survey)	*N* = 30 000 *n* = 1686 with endometriosis diagnosis and symptoms before diagnosis	Dec 2018‐Jan 2019	Yes (age, ethnicity, origin, gyn history)	Female	Canada	List of symptoms***	Same list of symptoms*** data divided into ever experienced and currently experienced symptoms	Surgical (30.8%) Empirical (32.7%) Physician‐suspected (56.4%) (*all self‐reported*)	Mean: 5.4 years	Mean: 3.1 years	Mean: 2.3 years	Obstetrician (53.0%) Primary care physician (19.0%) General surgeon (17.0%) Infertility specialist (7.3%) Urologist (1.1%) Other specialist (2.5%)
Fernley et al. 2021	Observational cross‐sectional study (analysis of publicly accessible online autobiographical accounts)	*N* = 49 accounts	Jun 2019‐Aug2019	No	Female	Australia	Not specified	Not specified	Not specified	< 1 year (*n* = 4) 2–5 years (*n* = 12) > 6 years (*n* = 27) 11–20 years (*n* = 12)	Not available	Not available	Not specified
Zhang et al. 2021	Observational cohort study (retrospective chart review)	*N* = 198 *n* = 184 white patients *n* = 14 black patients	2015–2020	Yes (race)	Female	US	Not specified	Pelvic pain (87.3%), dysmenorrhea (72.5%).	Surgical	Mean: 40.6 ± 56.9 months White patients (Mean): 40.3 ± 56.8 months Black patients (Mean): 43.7 ± 60.9 months	Not available	Not available	Not specified
Pino et al. 2023	Observational cross‐sectional study (online survey)	*N* = 940 *n* = 689 symptomatic and completed questionnaire	Oct 2014‐Jun 2019	Yes (age, origin, education, gyn history)	Female	Italy	Dysmenorrhea, dyspareunia, pelvic pain	Severe pain symptoms, 654 out of 689 patients (93.7%) scored at least 7 on the NRS scale when the pain intensity was at its worst in their lifetime	Surgical (+ histology, *n* = 344) Imaging (US/MRI, *n* = 332) Clinical evaluation (*n* = 17)	Mean: 11.4 ± 7.7 years 9–19 years old: 14.8 years (IQR: 10,19) 20–30 years old: 6.9 years (IQR: 1, 11) > 30 years old: 2.9 years (IQR: 0.5, 3) Further sub‐groups in full text (number of symptoms (1, 2 or 3) and severity of symptoms (< 7 or higher or equal than 7))	Not available	Not available	Not specified
Aubrey et al. 2023	Observational cross‐sectional study (consultation survey)	*N* = 84 *n* = 57 included for diagnostic delay assessment	Jun 2017‐Jun 2019	Yes (age, origin, BMI, occupation, gyn history)	Not specified	France	Not specified	Dysmenorrhea, abdominal pain outside of menses, transit disorder, rectorrhagia, pain on defecation, hematuria, dysuria, dyspareunia, infertility	Surgical (+ histology), 2% Imaging (Ultrasound/MRI), 42%/56%	Median: 12 years (min: 0, max: 33) Subgroup analysis by region and physician specialty in full text	Median: 4 years (min: 0, max: 27) Subgroup analysis by region and physician specialty in full text	Median: 8 years (min: 0, max: 33) Subgroup analysis by region and physician specialty in full text	Gynaecologist (*n* = 46) General practitioner (*n* = 11)
Di Vasta et al. 2018	Observational cross‐sectional study (online survey)	*N* = 402 *n* = 270 adolescents replied to diagnostic delay *n* = 101 adults replied to diagnostic delay	Nov 2012–Mar2016	Yes (age, race, BMI, education, gyn history)	Females	US	Pain, infertility	Pelvic pain, abdominal pain, dysmenorrhea, urinary or bowel movement habit changes, gastrointestinal symptoms	Surgical	Median in adolescents: 2 years (min: 0, max: 7) Median in adults: 5 years (min: 0, max: 26)	Median in adolescents: 1 year (min: 0, max: 7) Median in adults: 1 year (min: 0, max: 17)	Not available	Not specified

* Pelvic pain; Pelvic mass; Painful periods; Heavy periods; Infertility; Ovarian cyst; Painful intercourse; Pain on opening bowels; Bleeding from back passage when opening bowels; Bowel upset e.g.: constipation, diarrhoea; Pain on passing urine; Blood in urine; Other urinary problems.

** Back pain; Bloating; Blood in the stool; Blood in the urine; Chest pain; Constipation; Cramping; Cysts; Diarrhea; Dysmenorrhea; Dyspareunia; Excessive bleeding; Fatigue; Flank pain; Frequent urination; Headache; Infertility; Joint pain; Leg pain; Nausea; Painful bowel movements; Painful urination; Pelvic pain; Rectal pain; Weight gain.

*** Menstrual pelvic pain or cramping; Non‐menstrual pelvic pain or cramping; Dyspareunia; Fatigue, weariness, or anemia; Heavy menstrual bleeding; Excessive or irregular bleeding (e.g., spotting between periods); Passage of clots; Irregular periods (timing and/or duration); Pelvic pressure; Lower back pain; General abdominal pain; Constipation, bloating, or diarrhea; Difficulty having bowel movement; Frequent urination or urinary urgency; Infertility; Depressed feelings or mood swings; Dizziness during period; Anxiety or stress; Complications during pregnancy and labour.

Despite variations in study methodologies, settings and diagnostic methods, there was minimal heterogeneity observed among the populations studied. Where specified, the majority of included participants were white, highly educated women, aged 30 years or older and presented with multiple symptoms such as pelvic pain, infertility, dysmenorrhea and menstrual or cycle‐related gynaecological problems leading to diagnosis or at the time of study conduct. If information was available, focus was exclusively on study populations with a female gender identity.

**FIGURE 2:**
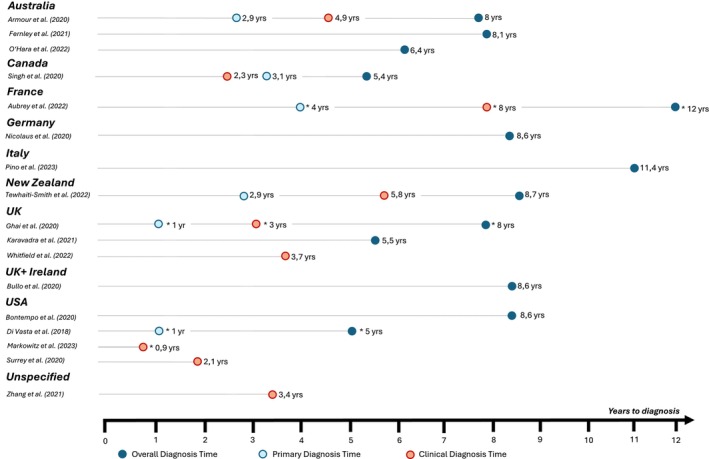
Diagnosis time distributions per country and publication included in this systematic review. The average time to diagnosis is presented as the mean. Instances where the median diagnosis time is reported are marked with an asterisk (*).

The data extraction revealed variations in diagnosis times ranging from 0.3 to 12 years. The overall time to diagnosis ranged from 5 to 12 years, the primary time to diagnosis ranged from 1 to 4 years and the clinical time to diagnosis ranged from 0.3 to 8.6 years. More details on the reported time to diagnosis per country can be found in Figure 2. Due to the difference in the interpretation of time to diagnosis based on the study design, it was decided to report results for cross‐sectional and cohort studies separately.

### 3.3 Cross‐Sectional Studies

Thirteen publications investigated time to diagnosis using a cross‐sectional study design, wherein the endometriosis diagnosis was predominantly self‐reported by participants, indicating that they were diagnosed by a physician or stated that their diagnosis was confirmed through, e.g., laparoscopy or other diagnostic methods that can only be performed by a healthcare practitioner. Among these studies, 11 reported a mean or median overall diagnosis time. The overall mean time to diagnosis ranged from 5.4 to 11.4 years, and the median time varied between 5 and 12 years. Six publications reported primary diagnosis time: The mean primary diagnosis time ranged from 2.9 to 3.1 years. The reported median primary diagnosis time varied between 1 and 4 years. Seven studies reported a clinical diagnosis time, which ranged from 2.3 to 8.6 years. Median clinical diagnosis time varied between 3 and 8 years.

### 3.4 Retrospective Observational Studies

Four publications used a retrospective cohort study design, in which time to diagnosis was identified via retrospective chart review and available diagnostic codes for endometriosis and endometriosis‐related symptoms. Due to the study design, we could only assess clinical diagnosis time for these publications. Mean clinical diagnosis time ranged from 2.1 to 3.7 years, and the median diagnosis time varied between 0.3 and 1.5 years.

### 3.5 Quality and Bias Assessment

Quality and bias assessment of the included studies was conducted using the CASP [17] and AXIS [18] tools for the retrospective cohort and the cross‐sectional studies, respectively. The studies were evaluated based on criteria such as study design, sample size, methodology and reporting quality. Common biases assessed included selection bias and reporting bias. The results of bias assessment are summarised in Appendix S4 (Tables S1 and S2). In summary, the assessment of eligible publications revealed a range from poor to fair quality for the cross‐sectional studies. For the retrospective cohort studies, assessment revealed a range from fair to good quality.

## 4 Discussion

### 4.1 Main Findings

We reviewed publications from January 1, 2018 to May 16, 2023, identifying 17 studies for data extraction. Despite a comprehensive search strategy, few studies addressed current diagnosis time, especially in specific populations like different ethnicities, gender identity groups, or non‐Western countries. This gap highlights the need for more research on diagnostic procedures and time to diagnosis across diverse endometriosis populations.

Diagnostic delays were still observed in this systematic review. Seventeen studies reported diagnosis times with varied definitions and study designs, making comparisons difficult. To address this, we categorised delays into three groups: overall, primary and clinical diagnosis times. The time to diagnosis showed significant variation, with mean and median times ranging from 0.3 to 12 years. This variation depended on how time to diagnosis was defined, the geographical location of the study and the characteristics of the study populations.

The overall time to diagnosis varied from 5 to 12 years. These findings align with previously published studies conducted on endometriosis and diagnostic delay between 2010 and 2018, reporting the time of first symptoms to diagnosis between 5 and 13 years [7, 12, 36, 37, 38, 39]. These findings and comparison with previous literature highlight that diagnostic delays have remained a persistent issue over the past decade despite advancements and increased awareness. Additionally, it highlights that improvements observed in the 1997 study [40] may not fully reflect the current landscape.

The primary diagnosis time highlights that patients wait 1–4 years in seeking medical attention after experiencing first symptoms. Factors contributing to this delay include a lack of awareness of symptoms [10] or symptom normalisation [41], fear of diagnosis, socioeconomic status, access to healthcare and cultural beliefs [42]. The variation in primary diagnosis times suggests that there are substantial differences in how quickly people seek medical help across regions and populations.

The clinical diagnosis time ranged from 0.3 to 8.6 years, indicating a considerable time between the initial consultation with a healthcare provider and receiving a confirmed diagnosis. Contributing factors may include misinterpretation of symptoms, symptom normalisation [43], lack of access to specialised diagnostic tools or expertise [44], healthcare system inefficiencies and diagnostic challenges due to complex or rare conditions [25, 45]. The wide range of clinical times to diagnosis could further suggest disparities in healthcare infrastructure, resources and expertise across different regions and healthcare settings. Despite existing (inter‐)national guidelines, healthcare systems and their qualities vary considerably between countries, and therefore, a more standardised approach is necessary in diagnosing endometriosis.

Five of the selected publications reported differences between patient subcategories, in which longer diagnosis times were seen in individuals who were overweight or obese compared to normal weight individuals [25], who were younger when presenting first symptoms [21, 26, 32], who had infertility compared to those without infertility [26], who had multiple comorbidities [26] and who were black compared to white individuals [35]. Another qualitative publication reported a twofold time to diagnosis for those who felt their pain not being taken seriously by the general practitioner [19]. Given that these findings were only observed in single studies, it is not possible to draw consistent conclusions. Further research on various patient characteristics and impact on diagnosis time is warranted to corroborate these observations.

Large differences were also seen between countries. The overall diagnosis time was the lowest in the United States and the highest in France. The primary diagnosis time was the lowest in the United Kingdom and the United States and the highest in France. The clinical diagnosis time was the lowest in the United States and the highest in France. Since multiple publications conducted in the United Kingdom, the United States and Australia were included in this review, we could compare diagnosis times reported within the different studies. We noticed large within‐country variations in diagnosis time among individuals living in these countries. In the United Kingdom, the overall diagnosis time ranged from 5.5 to 8 years, with clinical delays between 3 and 8.8 years. In the United States, the overall diagnosis time ranged from 5 to 8 years, with clinical delays between 2.1 and 3.4 years. In Australia, the overall diagnosis time varied from 6.4 to 8 years. Due to varying or unspecified descriptive information, the reasons for these differences remain unclear. Future studies need a consistent definition of diagnosis time, detailed study population descriptions and research into the interplay between patient factors, healthcare systems, provider practices and disease‐specific issues to address these disparities effectively.

### 4.2 Strengths and Limitations

This systematic review summarises the current status and existing challenges with regards to time to diagnose endometriosis and employed a thorough search strategy across PubMed and Embase, ensuring the inclusion of all relevant studies related to endometriosis and diagnostic delay. The review followed a pre‐defined and pre‐registered protocol outlining the study objectives, inclusion criteria and methods for data extraction and analysis. Adherence to established guidelines (i.e., PRISMA) ensured transparency and reproducibility of the review process. All studies from different geographical regions and diverse populations were considered. Another strength of this paper is the categorisation of time to diagnoses, which enhances comparability across various study designs and assessments, thereby increasing the robustness of our findings.

A couple of limitations should be considered when interpreting the results of this review. The majority of studies on endometriosis relied on cross‐sectional, online survey data and self‐reported, physician‐confirmed diagnoses. This approach can introduce selection bias, as participants in online surveys and forums might not represent the broader population affected by endometriosis [46]. They may have distinct characteristics, like a greater willingness to engage online or different symptom severity, skewing the results. Moreover, many studies lacked detailed baseline or disease characteristics of participants, limiting our ability to analyse diagnosis time in relation to these factors. The studies also included patients with diverse symptoms such as pelvic pain, infertility and other issues, complicating the analysis and generalisability of the findings. Additionally, most studies did not specify the specialties of diagnosing physicians, making it difficult to evaluate the influence of healthcare provider characteristics on diagnosis time. Despite focusing on papers from the last 5 years, some studies included data collected before 2018. Thus, their figures on diagnosis time might not accurately represent current timelines, potentially hiding any recent improvements in diagnosis speed. It should also be noted that the COVID‐19 pandemic, particularly for the studies assessing time to diagnosis from 2020 onwards [25, 34, 35], likely influenced diagnosis time due to restricted healthcare access and resource reallocation. The challenges posed by the pandemic may have contributed to longer diagnostic intervals, which should be considered when interpreting the findings. However, despite including study data during the COVID‐19 pandemic, the diagnosis times reported in the corresponding publications [25, 34, 35] were within the overall ranges, as observed across the other publications. Lastly, the quality assessment of eligible publications varied from poor to good, reflecting differences in study methodologies, reporting standards and potential biases. The generalisability of findings is restricted in some cases due to small sample sizes. This variability highlights the need to critically evaluate the evidence base when interpreting the review's findings.

### 4.3 Interpretation

Despite medical advancements and awareness campaigns, this systematic review confirms that diagnostic delays persist across healthcare settings, challenging timely patient care. Evidence suggests minimal improvement in diagnosis times in recent years, and the overall time to diagnosis remains high. Delays involve both patients and clinicians, but this review indicates that clinical diagnosis times are more significant than primary times. This highlights a need to raise awareness about endometriosis among healthcare providers, improve access to diagnostic resources and further research early diagnostic markers or interventions.

## 5 Conclusion

In conclusion, this systematic review highlights the ongoing issue of diagnostic delay in endometriosis. Both patients and physicians contribute to these delays, but longer delays typically occur on the physician's side. Our findings provide crucial insights into current diagnostic timelines but emphasise the need for a consistent definition of diagnosis time and a clear description of study populations. Understanding the factors contributing to these delays is essential for developing and implementing global interventions to reduce diagnostic delays effectively.

## Author Contributions

P.D.C., M.K. and S.v.S.: contributed to the statistical methodology of the study, development of the search strategy and criteria for inclusion and exclusion of studies, conducted the data analysis and synthesised the findings. They performed the quality assessment of included studies and interpreted the results in the context of existing literature, and contributed to preparing the figures and submission of the paper. M.K. and S.v.S.: were responsible for the systematic search and retrieval of relevant articles. P.D.C.: drafted the first version of the manuscript, and all authors contributed to the revision of the manuscript critically for important intellectual content, also contributed to the main concept and design of the study, and take responsibility for the integrity of the work as a whole and have given their approval for this version to be published.

## Ethics Statement

In conducting this systematic review, ethical approval was not required as the study involved the analysis and synthesis of data from previously published sources, without the involvement of new data collection or direct interaction with human subjects. This approach aligns with the standard practice in systematic reviews, which typically do not necessitate formal ethics approval. Nonetheless, all efforts were made to adhere to ethical guidelines, including ensuring the appropriate use and accurate citation of the original publications.

## Conflicts of Interest

The authors declare no conflicts of interest.

## Supporting information


**Appendix S1.** Search strings used in PubMed and Embase.
**Appendix S2.** Preferred Reporting Items for Systematic Reviews and Meta‐Analyses (PRISMA) checklist (2020).
**Appendix S3.** List of screened full texts and exclusion criteria (where applicable).
**Appendix S4.** Critical appraisal of observational studies using the CASP tool (Table S1). Critical appraisal of cross‐sectional studies using the AXIS tool (Table S2).

## Data Availability

Data sharing is not applicable to this article as no new data were created or analyzed in this study.

## References

[1] S. Davenport , D. Smith , and D. J. Green , “Barriers to a Timely Diagnosis of Endometriosis: A Qualitative Systematic Review,” Obstetrics and Gynecology 142, no. 3 (2023): 571–583. 37441792 10.1097/AOG.0000000000005255

[2] M. Zale , E. Lambert , M. D. LaNoue , and A. E. Leader , “Shedding Light on Endometriosis: Patient and Provider Perspectives on a Challenging Disease,” Journal of Endometriosis and Pelvic Pain Disorders 12, no. 2 (2020): 69–76.

[3] J. Frayne , T. Milroy , M. Simonis , and A. Lam , “Challenges in Diagnosing and Managing Endometriosis in General Practice: A Western Australian Qualitative Study,” Australian Journal of General Practice 52, no. 8 (2023): 547–555. 37532442 10.31128/AJGP-10-22-6579

[4] S. Matsuzaki , M. Canis , J.‐L. Pouly , B. Rabischong , R. Botchorishvili , and G. Mage , “Relationship Between Delay of Surgical Diagnosis and Severity of Disease in Patients With Symptomatic Deep Infiltrating Endometriosis,” Fertility and Sterility 86, no. 5 (2006): 1314–1316. 16978622 10.1016/j.fertnstert.2006.03.048

[5] W. P. Dmowski , R. Lesniewicz , N. Rana , P. Pepping , and M. Noursalehi , “Changing Trends in the Diagnosis of Endometriosis: A Comparative Study of Women With Pelvic Endometriosis Presenting With Chronic Pelvic Pain or Infertility,” Fertility and Sterility 67, no. 2 (1997): 238–243. 9022596 10.1016/S0015-0282(97)81904-8

[6] M. Mousa , M. Al‐Jefout , H. Alsafar , C. M. Becker , K. T. Zondervan , and N. Rahmioglu , “Impact of Endometriosis in Women of Arab Ancestry on: Health‐Related Quality of Life, Work Productivity, and Diagnostic Delay,” Front Glob Womens Health 2 (2021): 708410. 34816238 10.3389/fgwh.2021.708410 PMC8593935

[7] K. E. Nnoaham , L. Hummelshoj , P. Webster , et al., “Impact of Endometriosis on Quality of Life and Work Productivity: A Multicenter Study Across Ten Countries,” Fertility and Sterility 96, no. 2 (2011): 366–373.e8. 21718982 10.1016/j.fertnstert.2011.05.090 PMC3679489

[8] B. Swift , B. Taneri , C. M. Becker , et al., “Prevalence, Diagnostic Delay and Economic Burden of Endometriosis and Its Impact on Quality of Life: Results From an Eastern Mediterranean Population,” European Journal of Public Health 34, no. 2 (2024): 244–252. 38070492 10.1093/eurpub/ckad216 PMC10990517

[9] A. M. Soliman , M. Fuldeore , and M. C. Snabes , “Factors Associated With Time to Endometriosis Diagnosis in the United States,” Journal of Women's Health 26, no. 7 (2017): 788–797. 10.1089/jwh.2016.6003 28440744

[10] C. N. Simpson , C. M. Lomiguen , and J. Chin , “Combating Diagnostic Delay of Endometriosis in Adolescents Via Educational Awareness: A Systematic Review,” Cureus 13, no. 5 (2021): e15143. 34164243 10.7759/cureus.15143 PMC8214575

[11] M. van der Zanden , M. W. J. Arens , D. D. M. Braat , W. L. M. Nelen , and A. W. Nap , “Gynaecologists' View on Diagnostic Delay and Care Performance in Endometriosis in The Netherlands,” Reproductive Biomedicine Online 37, no. 6 (2018): 761–768. 30366841 10.1016/j.rbmo.2018.09.006

[12] G. Hudelist , N. Fritzer , A. Thomas , et al., “Diagnostic Delay for Endometriosis in Austria and Germany: Causes and Possible Consequences,” Human Reproduction 27, no. 12 (2012): 3412–3416. 22990516 10.1093/humrep/des316

[13] The American College of Obstetricians and Gynecologists , “Management of Endometriosis” 2010 [cited 2024 Mar 23], https://www.acog.org/clinical/clinical‐guidance/practice‐bulletin/articles/2010/07/management‐of‐endometriosis.

[14] C. M. Becker , A. Bokor , O. Heikinheimo , et al., “ESHRE Guideline: Endometriosis,” Human Reproduction Open 2022, no. 2 (2022): hoac009. 35350465 10.1093/hropen/hoac009 PMC8951218

[15] Royal Australian and New Zealand College of Obstetrics and Gynaecology (RANZCOG) , Endometriosis Clinical Practice Guideline (Melbourne, Australia: RANZCOG, 2021).

[16] National Institute for Health and Care Excellence (NICE) , Endometriosis: Diagnosis and Management. NICE Guideline [NG73] (London, UK: NICE, 2017). 29787038

[17] Critical Appraisal Skills Programme , “CASP Cohort Study Checklist” [cited 2022 Dec 13], https://casp‐uk.net/images/checklist/documents/CASP‐Cohort‐Study‐Checklist/CASP‐Cohort‐Study‐Checklist_2018.pdf.

[18] M. J. Downes , M. L. Brennan , H. C. Williams , and R. S. Dean , “Development of a Critical Appraisal Tool to Assess the Quality of Cross‐Sectional Studies (AXIS),” BMJ Open 6, no. 12 (2016): e011458. 10.1136/bmjopen-2016-011458 PMC5168618 27932337

[19] V. Ghai , H. Jan , F. Shakir , P. Haines , and A. Kent , “Diagnostic Delay for Superficial and Deep Endometriosis in the United Kingdom,” Journal of Obstetrics and Gynaecology 40, no. 1 (2020): 83–89. 31328629 10.1080/01443615.2019.1603217

[20] B. Karavadra , G. Thorpe , A. Stockl , and E. Morris , “Diagnostic Delay of Endometriosis in the United Kingdom; a Triphasic Mixed‐Methods Study: Top Scoring Abstracts of the RCOG Virtual World Congress 2021,” BJOG 128, no. Suppl 2 (2021): 4–281. 10.1111/1471-0528.16715 34096154

[21] E. Whitfield , M. E. Barclay , and G. Lyratzopoulos , “The Diagnostic Error in Medicine 14th Annual International Conference: Examining Variation in Time‐To‐Diagnosis and Symptomatic Presentation of Endometriosis,” Diagnosis 9, no. 2 (2022): 294–386. 35413161 10.1515/dx-2022-0024

[22] S. Bullo , ““I Feel Like I'm Being Stabbed by a Thousand Tiny Men”: The Challenges of Communicating Endometriosis Pain,” Health 24, no. 5 (2020): 476–492. 30782020 10.1177/1363459318817943

[23] A. C. Bontempo and L. Mikesell , “Patient Perceptions of Misdiagnosis of Endometriosis: Results From an Online National Survey,” Diagnosis 7, no. 2 (2020): 97–106. 32007945 10.1515/dx-2019-0020

[24] A. D. DiVasta , A. F. Vitonis , M. R. Laufer , and S. A. Missmer , “Spectrum of Symptoms in Women Diagnosed With Endometriosis During Adolescence vs. Adulthood,” American Journal of Obstetrics and Gynecology 218, no. 3 (2018): 324.e1–324.e11. 10.1016/j.ajog.2017.12.007 29247637

[25] M. A. Markowitz , M. Doernberg , H. J. Li , and Y. Cho , “Body Mass Index and Surgical Diagnosis of Endometriosis: Do Obese Patients Experience an Operative Delay?,” American Journal of Obstetrics and Gynecology 228, no. 3 (2023): S808–S809. 10.4103/gmit.gmit_137_23 PMC11626893 39660241

[26] E. Surrey , A. M. Soliman , H. Trenz , C. Blauer‐Peterson , and A. Sluis , “Impact of Endometriosis Diagnostic Delays on Healthcare Resource Utilization and Costs,” Advances in Therapy 37, no. 3 (2020): 1087–1099. 31960340 10.1007/s12325-019-01215-x PMC7089728

[27] M. Armour , J. Sinclair , C. H. M. Ng , et al., “Endometriosis and Chronic Pelvic Pain Have Similar Impact on Women, But Time to Diagnosis Is Decreasing: An Australian Survey,” Scientific Reports 10, no. 1 (2020): 16253. 33004965 10.1038/s41598-020-73389-2 PMC7529759

[28] N. Fernley , “That one doctor… Qualitative Thematic Analysis of 49 Women's Written Accounts of Their Endometriosis Diagnosis,” Journal of Endometriosis and Pelvic Pain Disorders 13, no. 1 (2021): 40–52.

[29] R. O'Hara , H. Rowe , and J. Fisher , “Managing Endometriosis: A Cross‐Sectional Survey of Women in Australia,” Journal of Psychosomatic Obstetrics and Gynaecology 43, no. 3 (2022): 265–272. 33050751 10.1080/0167482X.2020.1825374

[30] K. Nicolaus , L. Reckenbeil , D. Bräuer , R. Sczesny , H. Diebolder , and I. B. Runnebaum , “Cycle‐Related Diarrhea and Dysmenorrhea Are Independent Predictors of Peritoneal Endometriosis, Cycle‐Related Dyschezia Is an Independent Predictor of Rectal Involvement,” Geburtshilfe und Frauenheilkunde 80, no. 3 (2020): 307–315. 32139920 10.1055/a-1033-9588 PMC7056393

[31] G. Aubry , C. Bencharif , E. Vesale , et al., “Délais Diagnostiques et Parcours des Patientes Souffrant D'endométriose en France: une Étude Multicentrique,” Gynécologie Obstétrique Fertilité & Sénologie 51, no. 2 (2023): 117–122. 10.1016/j.gofs.2022.11.006 36423880

[32] I. Pino , G. M. Belloni , V. Barbera , et al., ““Better Late Than Never But Never Late Is Better”, Especially in Young Women. A Multicenter Italian Study on Diagnostic Delay for Symptomatic Endometriosis,” European Journal of Contraception & Reproductive Health Care 28, no. 1 (2023): 10–16. 36287190 10.1080/13625187.2022.2128644

[33] S. Singh , A. M. Soliman , Y. Rahal , et al., “Prevalence, Symptomatic Burden, and Diagnosis of Endometriosis in Canada: Cross‐Sectional Survey of 30 000 Women,” Journal of Obstetrics and Gynaecology 42, no. 7 (2020): 829–838. 10.1016/j.jogc.2019.10.038 32001176

[34] J. Tewhaiti‐Smith , A. Semprini , D. Bush , et al., “An Aotearoa New Zealand Survey of the Impact and Diagnostic Delay for Endometriosis and Chronic Pelvic Pain,” Scientific Reports 12, no. 1 (2022): 4425. 35292715 10.1038/s41598-022-08464-x PMC8924267

[35] W. Zhang , M. A. O'Brien , A. Frankel , and N. V. Clark , “Time to Diagnosis for Endometriosis by Race,” Journal of Minimally Invasive Gynecology 28, no. 11 (2021): S150.

[36] A. H. J. Staal , M. van der Zanden , and A. W. Nap , “Diagnostic Delay of Endometriosis in The Netherlands,” Gynecologic and Obstetric Investigation 81, no. 4 (2016): 321–324. 26742108 10.1159/000441911

[37] M. Moradi , M. Parker , A. Sneddon , V. Lopez , and D. Ellwood , “Impact of Endometriosis on Women's Lives: A Qualitative Study,” BMC Womens Health 14 (2014): 123. 25280500 10.1186/1472-6874-14-123 PMC4287196

[38] A. A. Graaff , T. M. D'Hooghe , G. A. J. Dunselman , C. D. Dirksen , L. Hummelshoj , and S. Simoens , “The Significant Effect of Endometriosis on Physical, Mental and Social Wellbeing: Results From an International Cross‐Sectional Survey,” Human Reproduction 28, no. 10 (2013): 2677–2685. 23847114 10.1093/humrep/det284

[39] X. T. Han , H. Y. Guo , D. L. Kong , J. S. Han , and L. F. Zhang , “Analysis of Characteristics and Influence Factors of Diagnostic Delay of Endometriosis,” Zhonghua Fu Chan Ke Za Zhi 53, no. 2 (2018): 92–98. 29534377 10.3760/cma.j.issn.0529-567X.2018.02.005

[40] H. Cox , L. Henderson , N. Andersen , G. Cagliarini , and C. Ski , “Focus Group Study of Endometriosis: Struggle, Loss and the Medical Merry‐Go‐Round,” International Journal of Nursing Practice 9, no. 1 (2003): 2–9. 12588614 10.1046/j.1440-172x.2003.00396.x

[41] C. X. Chen , C. Shieh , C. B. Draucker , and J. S. Carpenter , “Reasons Women Do Not Seek Health Care for Dysmenorrhea,” Journal of Clinical Nursing 27, no. 1–2 (2018): e301–e308. 28681499 10.1111/jocn.13946 PMC5746430

[42] K. Ballard , K. Lowton , and J. Wright , “What's the Delay? A Qualitative Study of Women's Experiences of Reaching a Diagnosis of Endometriosis,” Fertility and Sterility 86, no. 5 (2006): 1296–1301. 17070183 10.1016/j.fertnstert.2006.04.054

[43] S. K. Agarwal , C. Chapron , L. C. Giudice , et al., “Clinical Diagnosis of Endometriosis: A Call to Action,” American Journal of Obstetrics and Gynecology 220, no. 4 (2019): 354.e1–354.e12. 10.1016/j.ajog.2018.12.039 30625295

[44] H. Grundström , P. Kjølhede , C. Berterö , and S. Alehagen , ““A Challenge”—Healthcare Professionals' Experiences When Meeting Women With Symptoms That Might Indicate Endometriosis,” Sexual & Reproductive Healthcare 7 (2016): 65–69. 26826048 10.1016/j.srhc.2015.11.003

[45] K. Young , J. Fisher , and M. Kirkman , “Women's Experiences of Endometriosis: A Systematic Review and Synthesis of Qualitative Research,” Journal of Family Planning and Reproductive Health Care 41, no. 3 (2015): 225–234. 25183531 10.1136/jfprhc-2013-100853

[46] A. A. Graaff , C. D. Dirksen , S. Simoens , L. Hummelshoj , T. M. D'Hooghe , and G. A. Dunselman , “Quality of Life Outcomes in Women With Endometriosis Are Highly Influenced by Recruitment Strategies,” Human Reproduction 30, no. 6 (2015): 1331–1341. 25908657 10.1093/humrep/dev084

