# Supplementary material for: Time to Diagnose Endometriosis: Current Status, Challenges and Regional Characteristics—A Systematic Literature Review
Source: BJOG. 2024 Oct 7;132(2):118–30. doi: 10.1111/1471-0528.17973 (PMC11625652; doi:10.1111/1471-0528.17973)
Supplement: Supplementary file 1 — Appendix S1. Search strings used in PubMed and Embase. Appendix S2. Preferred Reporting Items for Systematic Reviews and Meta‐Analyses (PRISMA) checklist (2020). Appendix S3. List of screened full texts and exclusion criteria (where applicable). Appendix S4. Critical appraisal of observational studies using the CASP tool (Table S1). Critical appraisal of cross‐sectional studies using the AXIS tool (Table S2). [file BJO-132-118-s001.zip › 3_DeCorte_DiagnosticDelay_Appendix2_TableS1_ListOfFullTexts.docx]

**Appendix 2. Table S1. List of screened full texts and exclusion criteria (where applicable).**

| **#** | **Bibliographic Information of Screened Full Texts** | **Included (Y/N)** | **Exclusion Criteria*** |
| --- | --- | --- | --- |
| 1 | Bullo S. "I feel like I'm being stabbed by a thousand tiny men": The challenges of communicating endometriosis pain. Health (London). 2020 Sep;24(5):476-492. doi: 10.1177/1363459318817943. Epub 2019 Feb 19. PMID: 30782020 | Y | NA |
| 2 | Tewhaiti-Smith J, Semprini A, Bush D, Anderson A, Eathorne A, Johnson N, Girling J, East M, Marriott J, Armour M. An Aotearoa New Zealand survey of the impact and diagnostic delay for endometriosis and chronic pelvic pain. Sci Rep. 2022 Mar 15;12(1):4425. doi: 10.1038/s41598-022-08464-x. PMID: 35292715. | Y | NA |
| 3 | Markowitz MA, Doernberg M, Li HJ, Cho Y. Body mass index and surgical diagnosis of endometriosis: do obese patients experience an operative delay?. American Journal of Obstetrics and Gynecology, 2023; 228(3):808-809, Supplement, ISSN 0002-9378. doi: 10.1016/j.ajog.2022.12.036. | Y | NA |
| 4 | Nicolaus K, Reckenbeil L, Bräuer DM, Sczesny R, Diebolder H, Runnebaum IB. Cycle-related Diarrhea and Dysmenorrhea are Independent Predictors of Peritoneal Endometriosis, Cycle-related Dyschezia is an Independent Predictor of Rectal Involvement. Geburtshilfe und Frauenheilkunde. 2020;80:307 - 315. doi: 10.1055/a-1033-9588. | Y | NA |
| 5 | Ghai V, Jan H, Shakir F, Haines P, Kent A. Diagnostic delay for superficial and deep endometriosis in the United Kingdom. J Obstet Gynaecol. 2020 Jan;40(1):83-89. doi: 10.1080/01443615.2019.1603217. Epub 2019 Jul 22. PMID: 31328629. | Y | NA |
| 6 | Karavadra B, Thorpe G, Stockl A, Morris E. Diagnostic delay of endometriosis in the United Kingdom; a triphasic mixed-methods study. Special Issue:Top Scoring Abstracts of the RCOG Virtual World Congress 2021. BJOG. 2021 Jun;128(52):4-7. doi: 10.1111/1471-0528.16715. | Y | NA |
| 7 | Armour M, Sinclair J, Ng CHM, Hyman MS, Lawson K, Smith CA, Abbott J. Endometriosis and chronic pelvic pain have similar impact on women, but time to diagnosis is decreasing: an Australian survey. Sci Rep. 2020 Oct 1;10(1):16253. doi: 10.1038/s41598-020-73389-2. PMID: 33004965. | Y | NA |
| 8 | Whitfield E, Barclay ME, Lyratzopoulos G. Examining Variation in Time-to-diagnosis and Symptomatic Presentation of Endometriosis. Diagnosis. 2022;9(2):eA1–eA93. doi: 10.1515/dx-2022-0024. | Y | NA |
| 9 | Surrey E, Soliman AM, Trenz H, Blauer-Peterson C, Sluis A. Impact of Endometriosis Diagnostic Delays on Healthcare Resource Utilization and Costs. Adv Ther. 2020 Mar;37(3):1087-1099. doi: 10.1007/s12325-019-01215-x. Epub 2020 Jan 20. PMID: 31960340. | Y | NA |
| 10 | O'Hara R, Rowe H, Fisher J. Managing endometriosis: a cross-sectional survey of women in Australia. J Psychosom Obstet Gynaecol. 2022 Sep;43(3):265-272. doi: 10.1080/0167482X.2020.1825374. Epub 2020 Oct 13. PMID: 33050751. | Y | NA |
| 11 | Bontempo AC, Mikesell L. Patient perceptions of misdiagnosis of endometriosis: results from an online national survey. Diagnosis (Berl). 2020 May 26;7(2):97-106. doi: 10.1515/dx-2019-0020. PMID: 32007945. | Y | NA |
| 12 | Singh S, Soliman AM, Rahal Y, Robert C, Defoy I, Nisbet P, Leyland N. Prevalence, Symptomatic Burden, and Diagnosis of Endometriosis in Canada: Cross-Sectional Survey of 30 000 Women. J Obstet Gynaecol Can. 2020 Jul;42(7):829-838. doi: 10.1016/j.jogc.2019.10.038. Epub 2020 Jan 27. PMID: 32001176. | Y | NA |
| 13 | Fernley N. That one doctor. . . Qualitative thematic analysis of 49 women’s written accounts of their endometriosis diagnosis. Journal of Endometriosis and Pelvic Pain Disorders. 2021;13(1):40-52. doi:10.1177/2284026520984366. | Y | NA |
| 14 | Zhang W, O'Brien MA, Frankel A, Clark NV. Time to Diagnosis for Endometriosis by Race. Journal of Minimally Invasive Gynecology. 2021;28(11):150. doi: 10.1016/j.jmig.2021.09.297. | Y | NA |
| 15 | Pino I, Belloni GM, Barbera V, Solima E, Radice D, Angioni S, Arena S, Bergamini V, Candiani M, Maiorana A, Mattei A, Muzii L, Pagliardini L, Porpora MG, Remorgida V, Seracchioli R, Vercellini P, Zullo F, Zupi E, Vignali M; “Endometriosis Treatment Italian Club” (ETIC”. "Better late than never but never late is better", especially in young women. A multicenter Italian study on diagnostic delay for symptomatic endometriosis. Eur J Contracept Reprod Health Care. 2023 Feb;28(1):10-16. doi: 10.1080/13625187.2022.2128644. Epub 2022 Oct 26. PMID: 36287190. | Y | NA |
| 16 | Aubry G, Bencharif C, Vesale E, Oueld E, Dietrich G, Collinet P, Azais H, Canlorbe G. Délais diagnostiques et parcours des patientes souffrant d’endométriose en France : une étude multicentrique [Delays and pathways for patients with endometriosis in France: A multicenter study]. Gynecol Obstet Fertil Senol. 2023 Feb;51(2):117-122. French. doi: 10.1016/j.gofs.2022.11.006. Epub 2022 Nov 21. PMID: 36423880. | Y | NA |
| 17 | DiVasta AD, Vitonis AF, Laufer MR, Missmer SA. Spectrum of symptoms in women diagnosed with endometriosis during adolescence vs adulthood. Am J Obstet Gynecol. 2018 Mar;218(3):324.e1-324.e11. doi: 10.1016/j.ajog.2017.12.007. Epub 2017 Dec 13. PMID: 29247637. | Y | NA |
| 18 | Shukri MA, Riyami ASA, Ghafri WA, Gowri V. Are There Predictors of Early Diagnosis of Endometriosis Based on Clinical Profile? A Retrospective Study. Oman Medical Journal. 2023;38(1):e458. doi: 10.5001/omj.2023.35. | N | Outcome |
| 19 | Chan Sun M, Sunnoo K, Vencatachellum I. Learning to live with endometriosis: Findings from a phenomenological study among women in Mauritius, a state in the Indian Ocean. International Journal of Africa Nursing Sciences. 2022;17. doi: 10.1016/j.ijans.2022.100473. | N | Outcome |
| 20 | Frankel LR. A 10-Year Journey to Diagnosis With Endometriosis: An Autobiographical Case Report. Cureus. 2022 Jan 17;14(1):e21329. doi: 10.7759/cureus.21329. PMID: 35186587; PMCID: PMC8849430. | N | Study design |
| 21 | Karavadra B, Thorpe G, Morris E, Semlyen J. A grounded theory exploration of the delays to diagnosis of endometriosis. BJOG. 2022. doi: 10.1111/1471-0528.5_17178. | N | Outcome |
| 22 | Moss KM, Doust J, Homer H, Rowlands IJ, Hockey R, Mishra GD. Delayed diagnosis of endometriosis disadvantages women in ART: a retrospective population linked data study. Hum Reprod. 2021 Nov 18;36(12):3074-3082. doi: 10.1093/humrep/deab216. PMID: 34610108. | N | Outcome |
| 23 | Ross WT, Snyder B, Stuckey H, Ross IR, McCall-Hosenfeld J, Harkins GJ, Smith CP. Gynaecological care of women with chronic pelvic pain: Patient perspectives and care preferences. BJOG. 2023 Apr;130(5):476-484. doi: 10.1111/1471-0528.17355. Epub 2022 Dec 11. PMID: 36457127. | N | Outcome |
| 24 | Van Niekerk L, Johnstone L, Matthewson M. Health-related quality of life in endometriosis: The influence of endometriosis-related symptom presence and distress. J Health Psychol. 2022 Dec;27(14):3121-3135. doi: 10.1177/13591053221085051. Epub 2022 Mar 27. PMID: 35341340. | N | Outcome |
| 25 | Gouesbet S, Kvaskoff M, Riveros C, Diard É, Pane I, Goussé-Breton Z, Valenti M, Gabillet M, Garoche C, Ravaud P, Tran VT. Patients' Perspectives on How to Improve Endometriosis Care: A Large Qualitative Study Within the ComPaRe-Endometriosis e-Cohort. J Womens Health (Larchmt). 2023 Apr;32(4):463-470. doi: 10.1089/jwh.2022.0323. Epub 2023 Jan 19. PMID: 36656556. | N | Outcome |
| 26 | Mohan D, Heidenreich S, Ramsay C, Saraswat L, Pirie D, Scotland G. PIH69 THE NEED FOR PERSON-CENTRED CARE IN ENDOMETRIOSIS TREATMENT: QUALITATIVE INSIGHTS AND DISCRETE CHOICE EXPERIMENT DEVELOPMENT. Value in Health. 2020. doi: 10.1016/j.jval.2020.04.463 | N | Outcome |
| 27 | Lim K, Fong YF. The clinical profile of young and adolescent women with laparoscopically diagnosed endometriosis in a singapore tertiary hospital. Taiwanese Journal of Obstetrics & Gynecology. 2017;56:181e183. doi: 10.1111/1471-0528.15132. | N | Outcome |
| 28 | Le Roux A, McCall J, Pudwell J, Pyper JS, Bougie O. Therapeutic journey of adolescents and young adults with severe dysmenorrhea and endometriosis. Journal of Endometriosis and Pelvic Pain Disorders. 2022;14(4):183–191. doi: 10.1177/22840265221116271. | N | Outcome |
| 29 | Roux AL, McCall J, Pudwell J, Pyper J, Bougie O. Therapeutic journey of adolescents with severe dysmenorrhea. Journal of Obstetrics and Gynaecology Canada. 2021;(43): 666. doi: 10.1016/j.jogc.2021.02.060. | N | Duplicate |
| 30 | Trutnovsky G, Plieseis C, Bjelic-Radisic V, BertholinyGalvez MC, Tamussino K, Ulrich D. Vulvodynia and chronic pelvic pain in a gynecologic outpatient clinic. J Psychosom Obstet Gynaecol. 2019 Sep;40(3):243-247. doi: 10.1080/0167482X.2018.1477753. Epub 2018 May 31. PMID: 29848143. | N | Outcome |
| 31 | Marqués Ruiz A, Cámara Baeza S, Sánchez Santos Y. A new reported case of ileocecal infiltrative endometriosis, a disease which is probably underdiagnosed. Rev Esp Enferm Dig. 2018 Dec;110(12):835. doi: 10.17235/reed.2018.5732/2018. PMID: 30238758. | N | Study design |
| 32 | Anees A, Siddique K, Abouzeid H, Titi S. A Rare Case of Intestinal Low-Grade Endometrial Stromal Sarcoma With Glandular Differentiation and Associated Endometriosis. Cureus. 2021 May 2;13(5):e14801. doi: 10.7759/cureus.14801. PMID: 34123602; PMCID: PMC8191851. | N | Study design |
| 33 | Karavadra B, Morris E. A mixed Methods exploration of the delays to diagnosis of endometriosis in the United Kingdom: A triphasic study. BJOG. 2020. doi: 10.1111/1471-0528.16274. | N | Duplicate |
| 34 | Karavadra B, Morris E. Exploring the delays to diagnosis of endometriosis in the United Kingdom; a triphasic mixed-methods study. Human Reproduction. 2020. | N | Duplicate |

*Exclusion was non-exhaustive, i.e., one criterion was sufficient for exclusion, even though multiple criteria might have been applicable.
NA: not applicable.
