# Supplementary material for: Time to Diagnose Endometriosis: Current Status, Challenges and Regional Characteristics—A Systematic Literature Review
Source: BJOG. 2024 Oct 7;132(2):118–30. doi: 10.1111/1471-0528.17973 (PMC11625652; doi:10.1111/1471-0528.17973)
Supplement: Supplementary file 1 — Appendix S1. Search strings used in PubMed and Embase. Appendix S2. Preferred Reporting Items for Systematic Reviews and Meta‐Analyses (PRISMA) checklist (2020). Appendix S3. List of screened full texts and exclusion criteria (where applicable). Appendix S4. Critical appraisal of observational studies using the CASP tool (Table S1). Critical appraisal of cross‐sectional studies using the AXIS tool (Table S2). [file BJO-132-118-s001.zip › DeCorte_DiagnosticDelay_AppendixS1_SearchStrings.docx]

Appendix S 1. Search strings used in PubMed and Embase.

| **Database** | **Search string** |
| --- | --- |
| PubMed | ((("Delayed Diagnosis"[Mesh] OR "Missed Diagnosis"[Mesh] OR "Missed diagnosis"[tiab] OR "Missed diagnoses"[tiab] OR "Underdiagnosed"[tiab] OR "Undetected"[tiab] OR "Misdiagnosis"[tiab] OR "Silent endometriosis"[tiab] OR "Diagnosis time"[tiab] OR "Delayed Diagnosis"[tiab] OR "Delayed Diagnoses"[tiab] OR "Late Diagnosis"[tiab] OR "late diagnoses"[tiab] OR ((diagnosis[sh] OR diagnosis[tiab] OR diagnosed[tiab] OR diagnosing[tiab] OR diagnoses[tiab]) AND ("time factors"[mesh] OR delay[tiab] OR delayed[tiab] OR delays[tiab] OR delaying[tiab]))))) AND ((endometriosis[mesh] OR endometriosis[tiab] OR Endometrioses[tiab]))  Filters: Case Reports, Clinical Conference, Clinical Study, Clinical Trial, Clinical Trial, Phase I, Clinical Trial, Phase II, Clinical Trial, Phase III, Clinical Trial, Phase IV, Comparative Study, Controlled Clinical Trial, Multicenter Study, Observational Study, Pragmatic Clinical Trial, Randomized Controlled Trial, Years 2018-2023 |
| Embase | ('endometriosis'/exp OR 'adenomyosis externa' OR 'endometriosis' OR 'endometriosis externa') AND ('delayed diagnosis'/exp OR 'delayed diagnosis' OR 'diagnosis delay' OR 'diagnostic error'/de OR 'diagnosis error' OR 'diagnostic error' OR 'diagnostic errors' OR 'diagnostic mistake' OR 'diagnostical error' OR 'diagnostical mistakes' OR 'erroneous diagnosis' OR 'error, diagnostic' OR 'failure to diagnose' OR 'false diagnosis' OR 'faulty diagnosis' OR 'flawed diagnosis' OR 'incorrect diagnosis' OR 'misdiagnosis' OR 'mistaken diagnosis' OR 'wrong diagnosis' OR 'missed diagnosis'/exp OR 'missed diagnosis' OR 'missing diagnosis' OR 'diagnosis time'/exp OR 'diagnosis time' OR 'diagnostic time' OR 'time to diagnosis' OR 'underdiagnosis'/exp OR 'under diagnosis' OR 'under-diagnosis' OR 'underdiagnosis' OR 'late diagnosis'/exp OR undetected OR 'silent endometriosis') AND [2018-2024]/py AND [humans]/lim AND [abstracts]/lim AND ('case control study'/de OR 'case finding'/de OR 'case report'/de OR 'case study'/de OR 'clinical article'/de OR 'clinical audit'/de OR 'clinical trial'/de OR 'cohort analysis'/de OR 'comparative study'/de OR 'controlled clinical trial'/de OR 'controlled study'/de OR 'correlational study'/de OR 'cross sectional study'/de OR 'evidence based medicine'/de OR 'intervention study'/de OR 'interview'/de OR 'longitudinal study'/de OR 'major clinical study'/de OR 'medical record review'/de OR 'multicenter study'/de OR 'observational study'/de OR 'pilot study'/de OR 'prospective study'/de OR 'qualitative research'/de OR 'quality control'/de OR 'questionnaire'/de OR 'randomized controlled trial topic'/de OR 'retrospective study'/de OR 'sample size'/de OR 'semi structured interview'/de) AND ('article'/it OR 'article in press'/it OR 'conference abstract'/it OR 'preprint'/it) |
