# Supplementary material for: Time to Diagnose Endometriosis: Current Status, Challenges and Regional Characteristics—A Systematic Literature Review
Source: BJOG. 2024 Oct 7;132(2):118–30. doi: 10.1111/1471-0528.17973 (PMC11625652; doi:10.1111/1471-0528.17973)
Supplement: Supplementary file 1 — Appendix S1. Search strings used in PubMed and Embase. Appendix S2. Preferred Reporting Items for Systematic Reviews and Meta‐Analyses (PRISMA) checklist (2020). Appendix S3. List of screened full texts and exclusion criteria (where applicable). Appendix S4. Critical appraisal of observational studies using the CASP tool (Table S1). Critical appraisal of cross‐sectional studies using the AXIS tool (Table S2). [file BJO-132-118-s001.zip › DeCorte_DiagnosticDelay_AppendixS5_BiasAssessment_V02-00.docx]

**Table 1.** Critical appraisal of observational studies using the CASP tool

| **Publication** | **Surrey et al. 2020** | **Zhang et al. 2021** | **Whitfield et al. 2022** | **Markowitz et al. 2023** |
| --- | --- | --- | --- | --- |
| **Did the study address a clearly focused Issue?** | Yes | Yes | Yes | Yes |
| **Was the cohort recruited acceptably?** | Yes | Yes | Yes | Yes |
| **Was the exposure accurately measured to minimise bias?** | Yes | Yes | Yes | Yes |
| **Was the outcome accurately measured to minimise bias?** | Can’t say | Can’t say | Yes | Can’t say |
| **Have the authors identified all-important confounding factors?** | Can’t say | Can’t say | Can’t say | Can’t say |
| **Was the follow-up of the subjects complete enough?** | Yes | Yes | Yes | Yes |
| **Was the follow-up of the subjects long enough?** | Yes | Yes | Yes | Can’t say |
| **Do you believe in the results?** | Yes | Yes | Yes | Yes |
| **Can the results be applied to the local population?** | Yes | Can’t say | Yes | Can’t say |
| **Do the results of this study fit with other available evidence?** | Yes | Can’t say | Yes | Yes |
| **Are there implications of this study for practice?** | Yes | Yes | Yes | Yes |
| **Negative attributes** | **2** | **4** | **1** | **4** |
| **Conclusion** *(good, poor, fair)* | **Good** | **Fair** | **Good** | **Fair** |

The tool does not have a scoring system; we hence will use the following scoring to appraise the cohort studies: **≤2 negative attributes (no/can’t say) means good quality; 3–5 negative attributes means fair quality and ≥6 means poor quality**

**Table 2.** Critical appraisal of cross-sectional studies using the AXIS tool

| **Publication** | **Bullo et al. 2020** | **Tewhaiti-Smith et al. 2022** | **Nicolaus et al. 2020** | **Ghai et al. 2020** | **Karavadra et al. 2021** | **Armour et al. 2020** | **Aubrey et al. 2022** | **O’Hara et al. 2022** | **Bontempo et al. 2020** | **Singh et al. 2020** | **Fernley et al. 2021** | **Pino et al. 2023** | **DiVasta et al. 2018** |
| --- | --- | --- | --- | --- | --- | --- | --- | --- | --- | --- | --- | --- | --- |
| **Were the aims/objectives of the study clear?** | Yes | Yes | Yes | Yes | Yes | Yes | Yes | Yes | Yes | Yes | Yes | Yes | Yes |
| **Was the study design appropriate for the stated aim(s)?** | Yes | Yes | Yes | Yes | Yes | Yes | Yes | Yes | Yes | Yes | No | Yes | Yes |
| **Was the sample size justified?** | No | No | No | No | No | No | No | No | No | No | Yes | No | No |
| **Was the target/reference population clearly defined? (Is it clear who the research was about?)** | No | Yes | Yes | Yes | Can’t say | Yes | Yes | Yes | Yes | Yes | No | Yes | Yes |
| **Was the sample frame taken from an appropriate population base so that it closely represented the target/reference population under investigation?** | Can’t say | Can’t say | Yes | Can’t say | Can’t say | Can’t say | Can’t say | Can’t say | Can’t say | Yes | Can’t say | Can’t say | Can’t say |
| **Was the selection process likely to select subjects/participants that were representative of the target/reference population under investigation?** | No | No | Can’t say | Can’t say | Can’t say | Can’t say | Can’t say | Yes | Can’t say | Yes | Can’t say | Yes | Can’t say |
| **Were measures undertaken to address and categorise non-responders?** | No | No | Can’t say | No | No | No | No | No | Yes | Yes | No | No | No |
| **Were the risk factor and outcome variables measured appropriate to the aims of the study?** | Can’t say | Yes | Yes | Yes | Can’t say | Yes | Yes | Yes | Yes | Yes | Can’t say | Yes | Yes |
| **Were the risk factor and outcome variables measured correctly using instruments/measurements that had been trialled, piloted, or published previously?** | Can’t say | Yes | Yes | Yes | Can’t say | Yes | Yes | Yes | No | No | No | Yes | Yes |
| **Is it clear what was used to determine statistical significance and/or precision estimates? (e.g., p values, CIs)** | Yes | Yes | Yes | Yes | Can’t say | Yes | Yes | Yes | Yes | Yes | Yes | Yes | Yes |
| **Were the methods (including statistical methods) sufficiently described to enable them to be repeated?** | Yes | Yes | Yes | Yes | Yes | Yes | Yes | Yes | Yes | Yes | Yes | Yes | Yes |
| **Were the basic data adequately described?** | No | Yes | Yes | No | No | Yes | Yes | Yes | Yes | Yes | No | Yes | Yes |
| **Does the response rate raise concerns about non-response bias?** | Yes | Yes | Can’t say | No | Can’t say | Yes | Can’t say | Yes | Yes | Can’t say | Yes | Can’t say | Can’t say |
| **If appropriate, was information about non-responders described?** | No | Yes | Yes | No | No | No | Yes | No | Yes | No | No | No | No |
| **Were the results internally consistent?** | Yes | Yes | Yes | Yes | Yes | Yes | Yes | Yes | Yes | Yes | Yes | Yes | Yes |
| **Were the results of the analyses described in the methods presented?** | Yes | Yes | Yes | Yes | Yes | Yes | Yes | Yes | Yes | Yes | Yes | Yes | Yes |
| **Were the authors’ discussions and conclusions justified by the results?** | Yes | Yes | Yes | Yes | Yes | Yes | Yes | Yes | Yes | Yes | Yes | Yes | Yes |
| **Were the limitations of the study discussed?** | Yes | Yes | No | Yes | No | Yes | Yes | Yes | Yes | Yes | Yes | Yes | Yes |
| **Were there any funding sources or conflicts of interest that may affect the authors’ interpretation of the results?** | No | No | No | No | Can’t say | No | No | Can’t say | Can’t say | Yes | No | No | No |
| **Was ethical approval or consent of participants attained?** | Can’t say | Yes | Yes | No | Can’t say | Yes | Yes | Yes | Yes | Yes | No | Yes | Yes |
| **Negative attributes** | **11** | **5** | **5** | **7** | **14** | **6** | **5** | **6** | **6** | **5** | **11** | **5** | **6** |
| **Conclusion** *(good, poor, fair)* | **Poor** | **Fair** | **Fair** | **Fair** | **Poor** | **Fair** | **Fair** | **Fair** | **Fair** | **Fair** | **Poor** | **Fair** | **Fair** |

The tool does not have a scoring system; we hence will use the following scoring to appraise the cross-sectional studies: **≤4 negative attributes (no/can’t say) means good quality; 5–10 negative attributes means fair quality and ≥11 means poor quality**
